# Dendritic cell-derived exosomes as anti-cancer cell-free agents: new insights into enhancing immunogenic effects

**DOI:** 10.3389/fimmu.2025.1586892

**Published:** 2025-05-28

**Authors:** Tikhon Redkin, Victoria Turubanova

**Affiliations:** ^1^ Department of Genetics and Life Sciences, Sirius University of Science and Technology, Sirius, Russia; ^2^ Institute of Neuroscience, National Research Lobachevsky State University of Nizhny Novgorod, Nizhny Novgorod, Russia

**Keywords:** extracellular vesicles, dendritic cell-derived exosomes, immunotherapy, cancer treatment, immunogenic cell death

## Abstract

The secretome of immune cells is currently a major focus in both diagnostic and therapeutic contexts. Cell-free therapeutic agents attract even more attention in cancer immunotherapy research, as their properties are comparable to, and sometimes surpass, those of cell-based immunotherapy. This is particularly evident when dendritic cell-based vaccines are compared with dendritic cell-derived exosomes (dexosomes). However, there is still significant potential for further research and optimization. We propose incorporating immunogenic cell death stimuli into the production of dendritic cell-derived exosomes in order to improve their effectiveness as a cell-free anti-cancer treatment. In this review, we suggest a new strategy to enhance the immunogenic potential of dexosomes, as well as summarize and compare immunogenic proprieties of dendritic cells and dendritic cells-derived exosomes as anti-cancer agents.

## 1 Introduction

In recent decades, the concept of strengthening cancer patients’ immune systems has gained recognition. The idea of self-healing seems elegant and straightforward. The first instance of cancer immunotherapy was reported by William Coley in 1891, who noted that some cancer patients experienced spontaneous remission when they developed a Streptococcus skin infection, erysipelas. He then used an injectable mixture of live and inactivated bacteria, specifically Streptococcus pyogenes and Serratia marcescens, to develop a treatment (1). Later, in the 1950-1960s, the idea of anti-cancer immunoediting was introduced, setting the stage for further research on immunotherapy. It postulated that the immune system can both inhibit and stimulate tumor growth. In 1990, the Food and Drug Administration (FDA) approved a bacteria-based vaccine (alive/protein-containing) Bacillus Calmette-Guérin for bladder cancer, marking one of the first successful applications of an immunotherapy agent (2, 3). Sipuleucel-T—the first therapeutic cell-based anti-cancer vaccine—was approved in 2011 for metastatic castration-resistant prostate cancer. This milestone in cell immunotherapy paved the way for further advances in vaccine development. A pinnacle of achievement in cell immunotherapy has been the development of chimeric antigen receptor (CAR) T cells. CAR-T therapy has been used to treat various cancer types, including the first FDA-approved CAR-T drug tisagenlecleucel against acute lymphoblastic leukemia, axicabtagene ciloleucel (Yescarta) against non-Hodgkin lymphoma, and idecabtagene vicleucel (Abecma) against multiple myeloma (4).

Nevertheless, there are still several barriers to the widespread application of cell immunotherapy, such as its high cost and low accessibility, as well as immune system fatigue (5). Furthermore, cell immunotherapy is often regarded as an adjuvant, supportive treatment, or last resort when other clinical approaches prove insufficient or totally ineffective. A therapy based on membrane-bound vesicles could be the next step in the fight against cancer. As a result, researchers are becoming more interested in the idea of using immune cell derivatives as anti-cancer agents: these medications are potentially more effective, less ethically constrained, and more commercially viable.

One of the prospective therapeutic cell-free agents is dendritic cells-derived exosomes (DEX). In the first part of the article, we comprehensively describe dendritic cells as therapeutic anti-cancer agents. The second half focuses on a detailed description of DEX proprieties, including their advantages and limitations, and compares them with dendritic cells in the context of cancer treatment. Furthermore, a novel strategy to increase the immunogenic potential of DEXs is proposed.

## 2 Discovery of dendritic cells: a new step in understanding antigen presentation

Dendritic cells (DCs) show great promise as a therapeutic agent, progressing steadily through clinical trials and being increasingly incorporated into conventional therapies and combination strategies to treat different types of solid tumors (6, 7).

In 1973, Ralph Steinman and Zanvil Cohn were the first to identify DCs in the mouse spleen (8). The unique morphology of DCs and high expression of major histocompatibility complex (MHC) molecules led to their classification as a distinct type of antigen-presenting cells (APCs) (9, 10). In the late 1990s, DCs were established as essential for linking the innate and adaptive immune system via the presentation of processed antigens to T cells (11, 12). Since those findings had been reported, the idea of employing DCs to treat cancer emerged.

### 2.1 Advances and challenges in dendritic cell-based cancer immunotherapy: timeline of clinical research and application

The clinical exploration of DCs as therapeutic agents began with clinical trials focusing on their ability to present tumor antigens to T cells. Despite objective tumor response rates being typically moderate, below 15%, early research showed that DC-based vaccines could elicit immune responses in cancer patients. By the late 1990s, as antigen-presenting proprieties of DCs had been described, various clinical trials commenced to assess the efficacy of DCs in treating diverse malignancies, including melanoma and prostate cancer. For instance, a notable study published in the Journal of Experimental Medicine in 1999 illustrated that vaccination with peptide-pulsed mature DCs could expand specific cytotoxic T cells (CTLs) and induce regression of metastases in patients with advanced melanoma (13, 14).

The understanding of DCs’ roles has evolved over the years: researchers have shown that DC vaccines may increase overall survival rates in some patients, despite the initially limited responses to these vaccines. This has changed the way clinical effectiveness is evaluated. New strategies have emerged, including next-generation DC vaccines designed to enhance immunogenicity and combination therapies integrating DC vaccination with other cancer treatments (14–16).

As of the end of 2024, the ClinicalTrials.gov database by the National Library of Medicine included 69 active or recruiting clinical trials on DC-based or DC-targeted mono- or combined immunotherapy: 5 studies were in the early phase I, 40 – phase I, 37 – phase II, and none were in phase III or IV. The ‘Completed’ and ‘Terminated’ statuses were assigned to 204 and 48 studies, respectively (17).

Not registered in this database, a phase III anti-glioma DC vaccine trial has shown that autologous tumor lysate-loaded DC vaccine (DCVax-L) increased the median overall survival up to 19,3 months, achieving clinical and statistical significance in comparison with matched, contemporaneous external controls (18) (**Table 1**).

**Table 1:** Current state of dendritic cell-based vaccines clinical trials.

Tumor type	Treatment	Clinical protocol	References
Early phase 1			
High Risk Triple Negative Breast Cancer	Neoantigen DC vaccine	3 vaccinations summary: every 2 weeks; 2 booster shots on month 6 and month 12.	(19)
EBV-associated Nasopharyngeal Carcinoma	Autologous DC vaccine (KSD-101)	Injection once every 2 weeks for a total of 3-5 times: 3 obligatory injections and 2 boost injections if needed.	(20)
Phase 1			
Stage IV Pancreatic, Liver, Biliary Tract and Colorectal Cancer	Tumor lysate or carcinoembryonic antigen (CEA) derived DC vaccine	2 regimens: DC vaccine monotherapy on days 1, 8, 15, 29, 85, 141, 197, 253 and 309 or DC vaccine with booster of anti-VEGF/anti-PD-1: 1, 8, 15, 29, 85, 141, 197, 253 and 309 + Lenvatinib on day 43-77 and Nivolumab on day 43, 57 and 71	(21)
Multiple Myeloma (MM), Plasmacytoma	Post-CAR-T Ag-presenting and immune modifying DC vaccine	–	(22–24)
Recurrent or Progressive High-grade Glioma	Personalized DC vaccine (ZSNeo-DC1.1)	Complex DC vaccine injections cycles	(25)
Phase 2			
Pancreatic, Esophageal, Liver or Ovarian Cancer	IL15-transpresenting WT1-targeted DC vaccine	DC vaccine injection every 2 weeks (± 3 days) for a total of 6 vaccinations	(26–28)
Epithelial ovarian cancer (EOC)	Autologous tumor lysate-loaded autologous (cDC1)-based vaccine (XP-DC)	–	(29)
Endometrial cancer (EC)	Autologous DC vaccine (AdHER2DC)	Injections of DC-vaccine on day 1 of cycles 1-3 with optional up to 3 boost doses on day 1 of cycles 6, 9, 12 + Pembrolizumab and Lenvatinib	(30–33)
Colorectal Cancer	Autologous DCs loaded with autologous tumor homogenate	Injection of DC vaccine on day 1 + IL-2 daily for five days (days 3-7)	(34)
Advanced Ovarian, Fallopian Tube, or Primary Peritoneal Cancer	Multi-epitope folate receptor alpha-loaded DC vaccine + Pembrolizumab	–	(18)
Phase 3			
Glioblastoma	Autologous tumor lysate-loaded DC vaccine (DCVax-L)	DCVax-L on days 0, 10, and 20; in months 2, 4, 8 and months 12, 18, 24, and 30, with monthly temozolomide	(18)
Status “Completed” – 204 studies		Status “Terminated” – 48 studies	

### 2.2 Expanding role of dendritic cell vaccines in cancer therapy: mechanisms, combinatorial strategies, and future directions

Such a strong research interest in DCs is understandable and well-justified, as they have significant potential both as a standalone treatment and in combination with other immunotherapeutic cancer medications and chemotherapy (35, 36). It is clear today that DC vaccination is an important and expanding subject, with continuous research being done to increase its overall clinical efficacy and application versatility (37).

DCs are recognized as the most effective APCs, capable of inducing both innate and adaptive immune responses. They demonstrate exceptional proficiency in processing and presenting tumor antigens to T cells, which is crucial for initiating a robust immune response against cancer (38, 39). By presenting tumor-specific antigens, DCs can stimulate the production of cytotoxic T lymphocytes that specifically target and eliminate cancer cells (40). Clinical trials have repeatedly shown that DC-based treatments are safe, even for patients whose cancer has progressed. DCs are therefore a good choice for many patients who might not respond well to conventional therapies. DC-based therapy can promote immune responses that result in persistent remissions, offering hope for long-lasting results (41).

The effectiveness of treatment increases when DC-based immunotherapy is combined with other modalities, such as immune checkpoint inhibitors (ICIs). This combined approach has demonstrated encouraging results in various cancers, e.g., melanoma, non-Hodgkin lymphoma, and breast cancer, including enhanced immune responses and better clinical outcomes. However, the percentage of cancer patients who benefit from ICIs is still low (42). These advantages of DCs motivate researchers to create specific, targeted, and highly effective DC-based vaccines against different malignancies.

### 2.3 Dendritic cells-based vaccines: general practiсe and innovative strategies

To date, there is only one DC-based vaccine approved for clinical usage. Sipuleucel-T (Provenge) is an FDA-approved vaccine against metastatic castration-resistant prostate cancer. It is a DC vaccine based on autologous cancer cell stimulation, with prostatic acid phosphatase (PAP) as the main target. According to the clinical trial results, overall survival increased by nearly 6.5 times after vaccination compared to the placebo, and side effects, such as fever and chills, were rare (43). The clinical protocol involves *ex vivo* incubation of autologous DCs with a recombinant antigen protein combines PAP and GM-CSF. Yet, the vaccine application is limited by strict requirements for sterility, transportation, and manufacturing conditions. Only 50 centers around the world are capable of providing this kind of therapy (44).

The general principle of DC vaccine design is to induce the activation and maturation of the patient’s or donor’s DCs *ex vivo* with the primary tumor cell lysates, followed by their administration via subcutaneous injection. The first step is to obtain DC, for which the patient’s mobilized blood is the ideal choice. Mobilization is an inevitable step because the number of DC progenitors in the blood is low, about 0.2% of the total peripheral blood mononuclear cells. To increase the proportion of DCs and progenitor cells (e.g., CD34**^+^** hematopoietic stem cells), differentiation stimuli like granulocyte colony-stimulating factor (G-CSF) or granulocyte-macrophage colony-stimulating factor (GM-CSF) must be used (45). Next, DCs should be purified using leukapheresis or other similar methods. The remaining cells are then cultured in a specific cytokine environment (e.g., GM-CSF, IL-4, and/or TNF-α) to promote DC differentiation and maturation (46, 47). Once DCs reach a mature state, they are pulsed with specific tumor lysates and re-injected to the patient as a subcutaneous vaccine, with the follow-up for non-specific inflammation-like reactions and side effects, which are usually limited to fever, redness, local pain, and mild swelling (43, 48). Interestingly, DCs that are currently used as therapeutic agents can be pulsed not only with autologous tumor cell lysates or homogenates, but also with messenger RNA (mRNA), tumor-associated antigens (TAA), proteins, or modified nanoparticles, while cytokine cocktails are often used as maturation stimuli (49–53).

Some extraordinary examples of DC vaccines have been observed in clinical applications. The N.N. Petrov National Medical Research Center of Oncology (Saint-Petersburg, Russia) provides a promising example of treatment with a DC vaccine. There, DC vaccines are used as a routine method for melanoma: bone marrow-derived progenitors and photosensitizers are injected intratumorally and then activated by light. According to *in vitro* and animal studies, it might be immunogenic cell death of primary melanoma cells *in vivo* that activates DCs and stimulates specific immune response, but the results have not been published yet (54). The same scientific group also described a clinical case of effective DC vaccination against pediatric H3 K27M-mutant diffuse midline glioma. They observed a dynamic increase in the proportion of T-lymphocytes (CD3^+^CD19^-^) and natural killer (NK) cells (CD3^-^CD16^+^56^+^). There were steady trends in the proliferation of activated HLA-DR^+^ T helper cells (CD3^+^CD4^+^) and CTLs (CD3^+^CD8^+^). At the same time, the proportion of regulatory subpopulations—both CD3^+^CD4^+^CD25^high^CD127^low^ T regulatory (Treg) cells and CD3^+^CD16^+^56^+^ NKT cells—showed no significant growth. The profile of immune response is presented in this article but the fate of the patient is not clear (55). The use of the DC vaccine against soft tissue sarcoma, colon cancer, and kidney cancer is mentioned as well, but no data has been published in a scientific format (56).

As many strategies have emerged, some researchers are striving to improve well-described and, more importantly, clinically used approaches to lysate-pulsed DC vaccines. One fundamental principle that could be integrated into clinical practice is the targeted induction of immunogenic cell death (ICD) during lysate preparation. Immunogenic cell death is a type of regulated cell death characterized by cell stress, the release or surface exposure of damage-associated molecular patterns (DAMPs), activation of antigen-presenting cells, especially DCs, and, as a result, enhanced specific T-cell immune response. It has been shown that DCs pulsed with lysates of immunogenically killed cells are more effective than DCs pulsed with necrotic cell lysates both *in vitro* and *in vivo* (57–60). ICD was first described as immunogenic apoptosis in 2005 and was later expanded to include other types of cell death, such as ferroptosis, pyroptosis, necroptosis, and other modalities (61, 62).

### 2.4 Challenges in dendritic cell immunotherapy: overcoming tumor microenvironment barriers – focus on exosomes

The many advantages of DCs in cancer therapy include specific immune response activation via antigen presentation, long-lasting immunity, less harmful side effects compared to chemo- or radiotherapy, and high compatibility with other therapeutic approaches (37, 42, 63, 64). However, many limitations have yet to be overcome. Thus, the tumor immune microenvironment (TIME) has a strong immunosuppressive DC-targeted profile. The subset of DCs typically used in immunotherapy due to accessibility issues — monocyte-derived DCs from mobilized peripheral blood — have lower efficacy in anti-tumor response than conventional DCs, previously known as myeloid DCs derived from a common DC progenitor (65–67). As described earlier, mobilization of monocytes and hematopoietic stem cells is critical for DC vaccination because of the constraints of autologous transplantation (68, 69). Here, researchers and clinical practitioners face yet another problem: the immune system of cancer patients develops fatigue and exhaustion as a consequence of chemotherapy and radiotherapy, cytokine imbalances, long-term bone marrow injury, and comorbidities (70, 71). Furthermore, limited lymph node infiltration in tumor-bearing individuals contributes to immunosuppression. If professional APCs cannot effectively penetrate the lymph nodes, they may fail to activate the full potential of T cells, resulting in a weaker targeted anti-tumor response.

Altered chemokine expression, phenotypic changes, challenges in identifying target antigens for DC stimulation, the risk of both immunosuppression and overstimulation, and the weakened immune status of late-stage cancer patients all similarly affect treatment outcomes (72–77). To overcome these limitations, novel immunotherapeutic strategies must be developed. Among emerging options, DC-derived exosomes show particular promise as an innovative approach to immunotherapy.

## 3 Dendritic cell-derived exosomes – cell-free saviors?

### 3.1 Exosomes as nanoscale mediators: content and structure

Exosomes are nanosized extracellular vesicles, typically ranging from 30 to 150 nm in diameter. They can be secreted by various cell types both under normal conditions and in response to external stimuli or due to acquired impairments (78). Exosomes play a crucial role in intercellular communication by transporting proteins, lipids, and nucleic acids from donor to recipient cells. This unique capability makes exosomes promising candidates for targeted drug delivery systems in therapeutic applications. Exosomes are primarily composed of a lipid bilayer, proteins (including heat shock proteins (Hsp) and membrane transport proteins), and nucleic acids (mRNAs and microRNAs). The lipid bilayer protects the cargo from degradation and facilitates fusion with target cell membranes (79–81). Exosome uptake by recipient cells involves several mechanisms, including receptor-ligand interactions, membrane fusion, and endocytosis. The specific uptake pathway can vary depending on the type of recipient cell and the surface proteins present on the exosomes (79, 82, 83).

### 3.2 Dendritic cell-derived exosomes: mechanisms of antigen presentation

One of the most impressive properties of DEX surface membranes, contributing to their immunogenic potential, is the presence of markers involved in specific antigen processing and presentation. DC-derived exosomes were first described in the context of their immunological function in 1998. This discovery was made by Zitvogel et al., who demonstrated that MHC-II enriched exosomes could present peptide–MHC II complexes to T cells, thus activating specific immune responses (84, 85). Worth noting that MHC-expressing exosomes secreted from B lymphocytes were detected and described two years earlier, in 1996, by Raposo et al. who first reported that extracellular vesicles are able to present antigens to T cells (86).

Usually, DC-derived exosomes are 30–150 nm extracellular vesicles that transport and deliver molecular signals, carry a variety of receptors, and inhibit immune surveillance (87, 88). They can present TAA to T cells, promoting a robust immune response. Interestingly, DEXs have a stimulatory effect on APCs termed ‘cross-dressing’. Exosomes from DCs transfer MHC-peptide complexes directly to other APCs, enhancing their ability to stimulate T-cell responses against tumors (89–91). Studies indicate that exosomes derived from DCs can efficiently capture and present antigens, leading to improved activation of CD8^+^ T cells compared to direct antigen presentation by DCs.

The DEX specific content and surface profile can induce a T-cell immune response either directly or indirectly (87, 92). The indirect pathway involves antigen cross-presentation via the MHC-peptide complex on exosomes, followed by reprocessing of antigen in DCs. Then, DCs present newly recognized peptides to T cells. In contrast, the direct pathway implies immediate interaction between T cells and MHC complexes on the surface of the DEXs (88, 93, 94).

Interestingly, DEXs are able to make the tumor more attractive for immune cells. A variety of strategies was described for breast carcinoma, melanoma, and hepatocellular carcinoma (89, 95–97). DEXs contain a rich array of MHC molecules, both class I and II, and co-stimulatory signals crucial for effective T-cell activation. Furthermore, it has been found that DEX membranes are enriched with MHC molecules compared to DCs (98). Major surface molecules responsible for antigen presentation on DCs are also found on DEX: MHC-I, MHC-II, CD80, CD86, CD40, etc. (87). Notably, co-stimulatory molecules B7.1 and B7.2, originally very important in T-cell response activation via different interactions, have also been identified on DEX. Additionally, intercellular adhesion molecule 1 (ICAM-1) has been shown to be essential for T-cell priming, as its expression as a co-stimulatory molecule is critical for the immunogenic properties of DEX in the anti-tumor immune response (92).

### 3.3 Dexosomes: next step in overcoming the limitations of DC-vaccines

**Figure 1:**
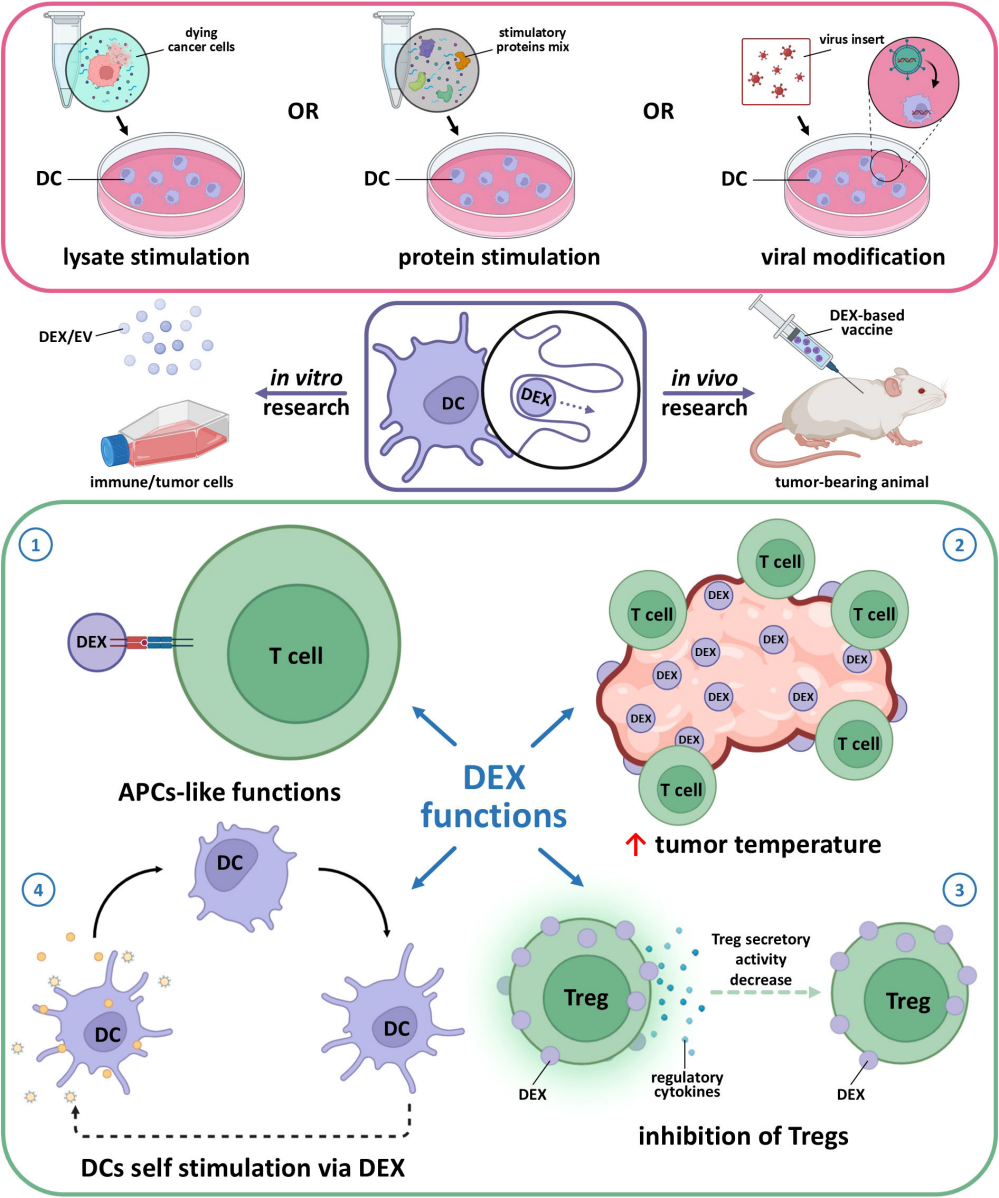
Main effects of dendritic cell-derived exosomes (DEX) obtained from mature stimulated dendritic cells. 1 – APC-like function assumes DEX interaction with T cells via MHC-TCR complex for antigen presentation as it happens in case of cellular (DC-T cell) contact. 2 – DEX are able to recruit immune cells to the tumor same as entice DC from TME to act as anti-tumor active cells. 3 – modified DEX (e.g. caring FasL, TNF-α) are able to suppress Treg pro-tumor activity via MHC-TCR interaction along with shifting the balance away from Treg dominance to effector T cells activation as DEX express co-stimulatory molecules. 4 – DEX express integrins, ICAM-1, and tetraspanins (CD9, CD63, CD81) which enable efficient binding to and uptake by other DCs, also DC-derived EVs are able to transfer peptide-MHC complex to recipient DCs, which then reprocess and present these antigens, amplifying the immune response.

Derived from cells, exosomes naturally have high biocompatibility, which makes them suitable for clinical applications (82) and a possible answer to the limitations of DC vaccine therapy. DEXs have a more targeted action: their sizes range from 30 to 150 nm, allowing them to migrate and internalize within lymph nodes. DEXs are also less susceptible to the TIME due to their stable composition and phenotype, which remains unaffected by immunosuppressive immune and tumor cells (87, 99–101). There are reports that DCs activated *in vitro* can switch into an immunosuppressive phenotype associated with a weaker immunotherapy effect (102, 103). In the TIME, DEXs are able to change pro-tumor immune cells into anti-tumor cells. Exosomes from activated DCs can induce a pro-inflammatory environment, essential for effective anti-tumor immunity. They achieve this by enhancing the expression of pro-inflammatory cytokines and promoting the maturation of other DCs, which in turn can stimulate T-cell responses against tumors (104–107). Exosomes influence the tumor microenvironment (TME) by altering the behavior of stromal cells, such as fibroblasts and immune cells. For instance, they can induce the secretion of cytokines and chemokines that recruit more immune cells to the tumor site, thereby creating an environment conducive to immune attack (108). Additionally, exosomes can inhibit the immunosuppressive functions of Tregs and myeloid-derived suppressor cells, further enhancing anti-tumor immunity (**Figure 1**) (109, 110). T cells can independently attract DEX via the LFA-1 molecule, potentially enhancing the immune response (111). The interaction of DEXs with CD4^+^ results in MHC I expression in the CD4^+^ population and facilitates CTL transition.

Importantly, exosomes can easily penetrate biological barriers such as the blood-brain barrier or blood-tumor barrier (112, 113). This could be leveraged in brain tumor treatment (114), as not all immunotherapeutic and chemotherapeutic drugs can penetrate these barriers to accumulate in the target tumor (115). For example, temozolomide, a standard chemotherapy drug for glioblastoma multiforme, only reaches about 20% of its blood concentration within the brain (115).

Romagnoli et al. have hypothesized that tumor cells treated with DEX could induce anti-tumor effects *de novo* and stimulate migration of both primary and modified immune cells and interact with them. Through a series of complex incubation steps using DC+tumor-derived exosomes, activated DC+T-cells, and interferon-γ (IFN-γ)-producing T-cell detection, it was elegantly demonstrated that tumor cells treated with DC-derived exosomes become more attractive targets for existing immune effector cells (89).

Furthermore, we should mention the economic benefits of DC-derived exosomes. During the manufacturing process, DEX can be easily stored at -80˚ C for 3–6 months, their homogeneity is sufficient for massive production, and as subcellular components, exosomes have significantly fewer ethical restrictions in clinical usage (92, 116–118). The main features and effects of DEXs are illustrated in **Figure 1**.

### 3.4 Challenges and considerations in dendritic cell-derived exosome production: standardization and optimization

There are currently no standardized guidelines or criteria for DEX manufacturing (119). While it is evident that DCs are the source of dexosomes, several key factors are overlooked. For instance, the subtype of DCs producing DEXs is often disregarded. Furthermore, methods for identifying intravesicular content are complex and costly. Thus, more aspects and methods should be reviewed to improve standardization, optimization, and applicability.

**Figure 2:**
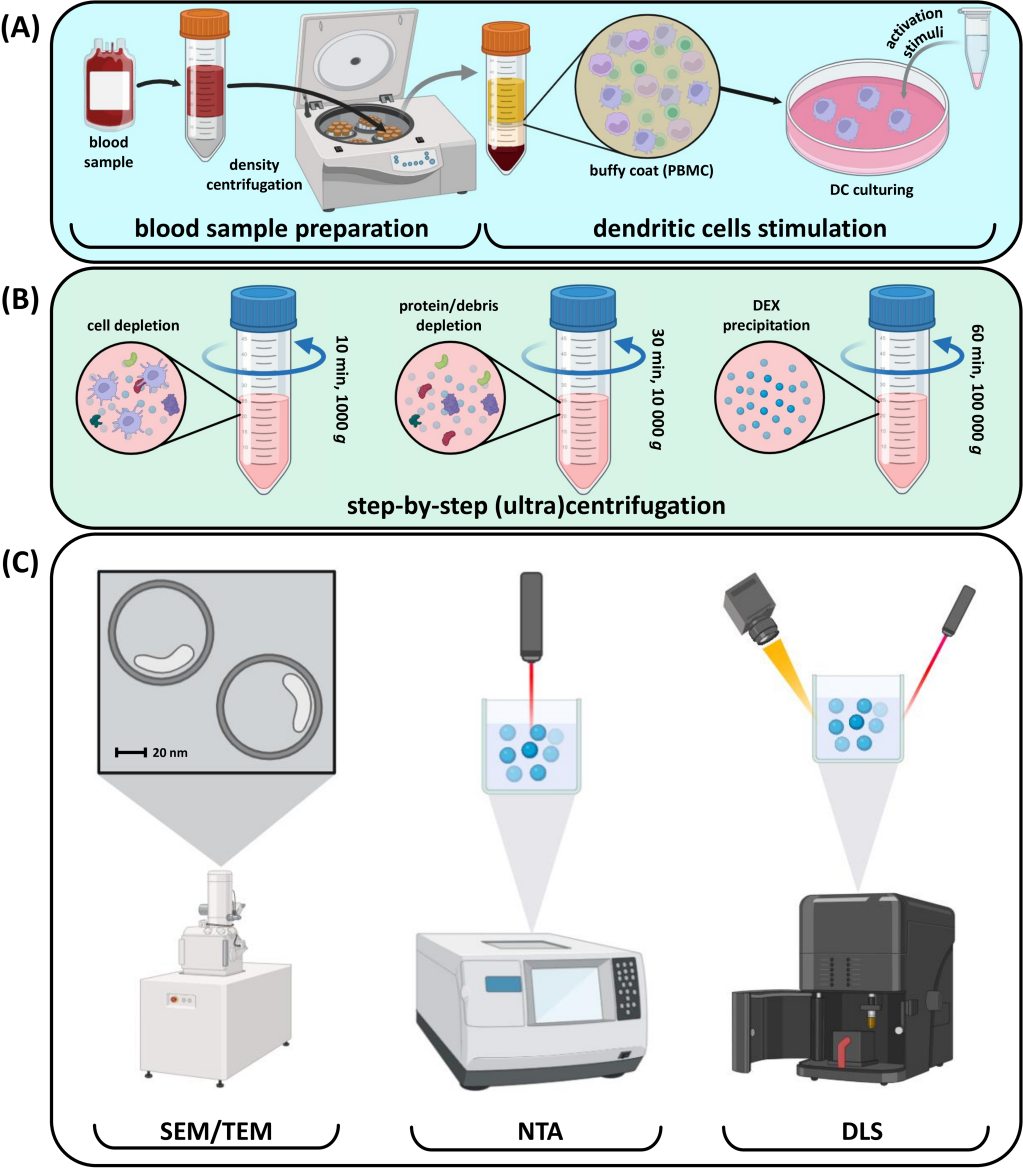
Common steps involved in DEX production and quality control. **(A)** Blood processing and DC stimulation; **(B)** Сonsecutive DEX purification with centrifugation; **(C)** Purified DEX size and quality control.

The common DEX production process begins with ex vivo DC cultivation and stimulation of their mature phenotype as one of the most critical points that determine the effectiveness of immune system activation by DEXs. The subsequent manipulations include the incubation of DCs with tumor lysates or proteins and several genetic, e.g., viral, modifications of DCs (**Figure 2A**). After exposure to antigenic stimuli, DEXs are collected from the culture media and washed several times before analysis (87, 120) (**Figure 2B**). Typical DEX analysis includes several techniques (**Figure 2C**) (121–123). Scanning electron microscopy is used to assess the morphology of extracellular vesicles, which is crucial for understanding their biological functions and potential applications in diagnostics and therapeutics. Nanoparticle tracking analysis allows for rapid and precise measurement of vesicle size and concentration. Dynamic light scattering helps assess biophysical properties of extracellular vesicles, while immunoblotting is used to valuate purity and detect different proteins and markers they carry.

*In vivo*-focused research includes additional methods like flow cytometry for phenotype detection and mass spectrometry for identifying internal DEX content (124). After purification, DEXs can be used as active vesicles in various applications.

Purity of exosomes is a critical factor in the context of both *in vitro* and *in vivo* studies. Inappropriate characterization of purified EVs might cause the lack of efficacy, false assays interpretation and additional immunological load on organism. So, there are several strategies to prevent the production of unearmarked EVs. On of the strategies include the usage of culture medium depleted of bovine serum exosomes. This method implies ultracentrifugation for serum-derived exosomes precipitation and following culture medium preparation without pelleted exosomes (125). Another strategy is based on the known size of DEX. To improve purity and separate exosomes from other vesicles and protein aggregates, density gradient ultracentrifugation could be used. The goal product typically located in one of the fractions depending on target size of the EV (126). Immunoaffinity isolation is a precise and high-selective method of DEX isolation. Magnetic beads coated with antibodies against exosome-specific surface markers (e.g., MHC-II molecules) could be used while selective DC-derived exosomes capturing (127).

To ensure the safety and efficacy of dendritic cell-derived exosomes for different patients, strict quality control measures throughout production should be implemented. Firstly, criteria for dendritic cell vaccination could be applied in the context of the ability to get the cells from the blood through the general volume of DC and DC precursors measurement. As the source of the DEX are dendritic cells, so the first criteria should be the ability to culture and stimulate autologous dendritic cells (128). The second criteria could be presented as a list of requirements for detailed EVs characterization (e.g. identity, purity, structure and sterility), functional compliance and storage (98, 129). Adhering to guidelines and regulations set forth by organizations like the International Society for Extracellular Vesicles (ISEV) and the European Network on Micro-vesicles and Exosomes in Health and Disease (ME-HaD) is another step to the clinical safety of DEX-based treatment (130).

The versatility of DEX remains an important but largely unexplored area of research. Most studies report obtaining DEXs from allogeneic DCs, and graft-versus-host reactions have not been described. This raises the question of HLA typing for DEX, which is another challenge researchers have to face. Overall, the use of DEXs derived from non-donor DCs is still under discussion. Addressing these questions will require approaches rooted in autoimmune research and allogeneic transplantation algorithms.

### 3.5 Current status and preclinical advancements in DEX-based anti-cancer therapy

As of 2024, the clinical trials database (https://clinicaltrials.gov/) lists only two clinical trials related to anti-cancer treatment with DC-derived exosomes. The first is a completed phase II clinical trial for unresectable non-small cell lung cancer. However, the results have not yet been published and no data on survival or effectiveness are available. Still, the trial included a well-documented clinical protocol involving stimulatory proteins for DC activation, cyclophosphamide treatment, and intradermal injections of DEX—administered weekly for four weeks and followed by injections every two weeks for six weeks (131). The second study was completed at the end of 2023 and investigated DEX therapy for bladder cancer but details on the DEX design and clinical protocol are not available (131). Notably, most clinical studies focus on the role of exosomes of different origin as diagnostic molecules for various diseases.

In contrast to the limited number of clinical trials, likely due to the novelty of this approach, numerous fundamental and pre-clinical studies have been described. Thus, exosomes enriched with chaperones proved an effective way of glioma treatment in mice. The chaperone-rich lysate contained at least four chaperone proteins, including Hsp70 and Hsp90, calreticulin, and glucose-regulated protein 94 (GRP94), which made the treatment more effective (132). Lu et al. showed that DEX derived from α-fetoprotein-expressing DCs activated a specific anti-tumor response against hepatocellular carcinoma and increased the number of CD8+ T cells in the TME. Furthermore, CD8+ cells gradually increased the level of immunostimulatory cytokines, such as IL-2 and INF-γ, while also reducing CD25+ expression and CD25+FoxP3+ the proportion of Tregs, as well as lowering IL-10 and TGF-β in tumor sites (97). Another DEX-based approach has been used for cervical cancer. This study presented a protocol for DEXs engineered via DC stimulation with the HPV early antigen 7 protein, the main target antigen for this type of tumors (133). In human gastric adenocarcinoma, the CTL immune response was induced using either tumor lysate or RNA to treat DCs, with tumor RNA being more effective. Exosomes were then obtained and injected as a vaccine. In a phase I clinical trial, DEXs derived from autologous plasmacytoid DCs have shown mixed effectiveness against melanoma. Only one patient in this study had a pronounced T-cell response, but researchers observed a high proportion of infiltrating NK cells and proposed that DEXs are important for NK activation (134). This finding was later supported by multiple studies and another phase I clinical trial (88, 135, 136). Combination of DEXs with anti-CTLA-4 ICI treatment was able to strongly enhance the efficacy of immune response (137). Using chimeric DEX-like exosomes for glioblastoma treatment in the orthotopic model, researchers found an increase in tumor-infiltrating T-cells, a better Ki-67response, and the maintenance of memory T cells (138). For gastric cancer, hybrid exosomes were engineered. DEXs modified with a chemotherapeutic agent induced an effective anti-cancer immune response in a murine model (139).

### 3.6 Immunogenic cell death as a strategy for to enhance dendritic cell-derived exosome-based cancer therapy

As described above, immunogenic cell death (ICD) is a form of regulated cell death. It is characterized by exposure to DAMPs released from dying tumor cells, followed by the activation of specific immunity (61, 140). We propose DEX therapy could benefit from employing core determinants of ICD. First, the cell should undergo stress adaptation as it leads to intracellular changes and shapes the immunogenic profile of cell death mediated by therapeutic modifications. The second determinant is that dying cells should be taken up by antigen-presenting DCs rather than macrophages, to promote an effective immune response. The third important point is the sufficient antigenicity of the dying cells. The last determinant is the adjuvant effect, which involves the release of DAMPs as immune-stimulating molecules (141).

**Figure 3:**
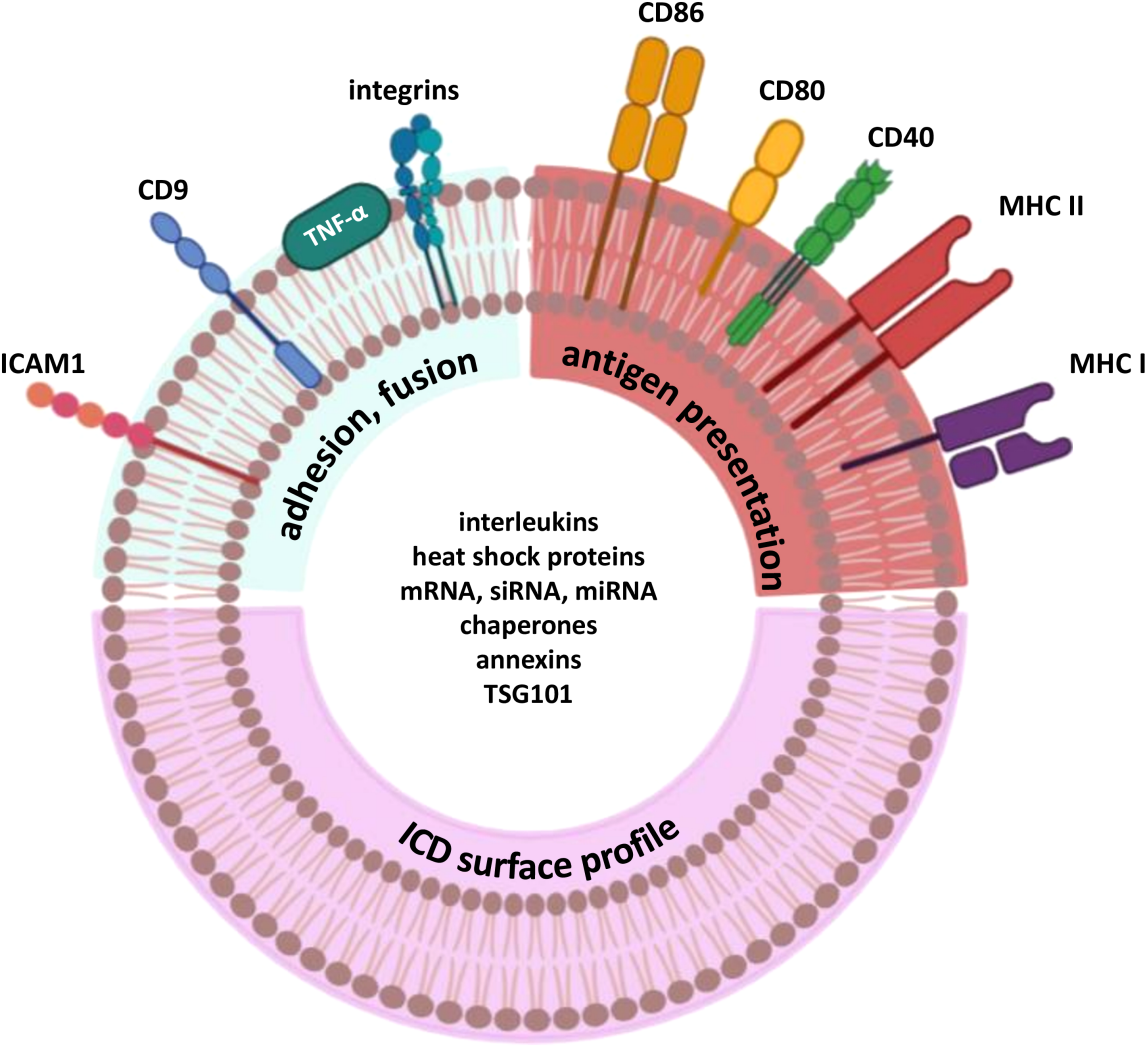
Probable composition of exosomes derived from dendritic cells pulsed with ICD stimuli.

With various methods of its induction available, ICD is an elegant cell death pathway to be used in research and development. This cell death modality can be activated using classical inducers, such as chemotherapeutic drugs, e.g., anthracyclines, cyclophosphamide, bortezomib, radiotherapy (142), or photodynamic therapy when immunogenic photosensitizers (PS) are used, e.g. porphyrazines, G-chlorin, and nanocarriers with PS (143–145). Interestingly, there are some rarely used methods of ICD induction, like cold atmospheric plasma (146) or natural compounds capsaicin and curcumin (147). Since immunogenic apoptosis was first described by Guido Kroemer’s group in 2005, the number of studies focusing on ICD has increased rapidly. As of 17th December, 2024, PubMed database includes 5,262 articles mentioning immunogenic cell death (61, 148). Given that immunogenic cell death markers include cell surface proteins, they may also be exposed on extracellular vesicles in a similar manner. We further suggest that DEXs could be enriched with immunogenic surface proteins, as this has been observed for MHC molecules (98). While the DEX classical phenotype has been described, there is no data on their inner and surface composition in the context of ICD-mediated DEX (**Figure 3**). As MHC and co-stimulatory molecules are common for the majority of DEX, some of inner molecules like Hsp and annexins might be detected as DAMPs which play a key role in immune system response during ICD-based activation (61, 87). On the other hand, the specific surface profile after ICD stimuli has not been clearly described which opens a wide area for the research of dendritic cell-derived exosomes in the context of therapy search.

**Figure 4:**
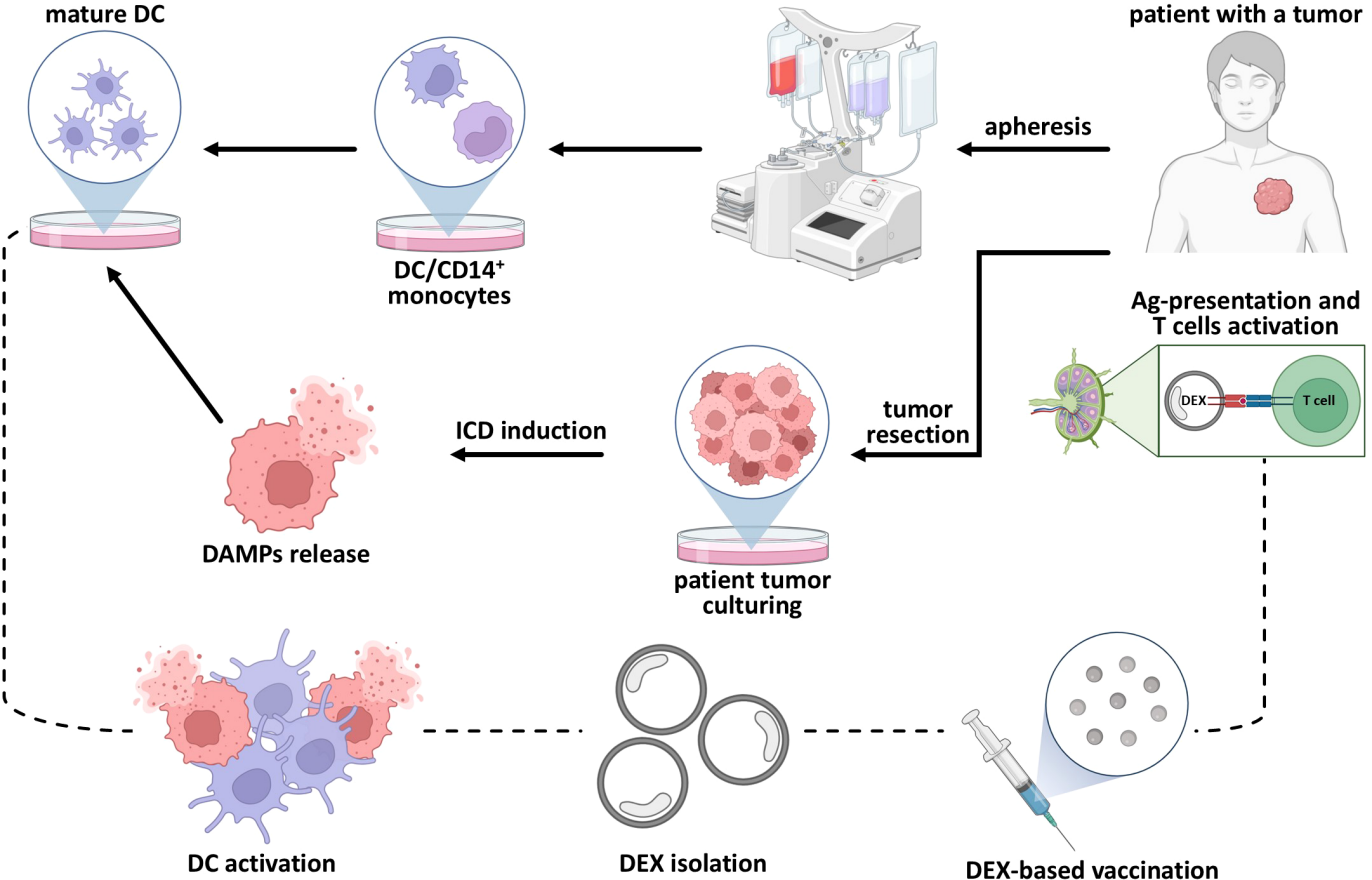
Combination of ICD and DEX-based vaccination as a strategy for anti-tumor treatment. Classical ICD maturation stimuli include immunogenically killed cells loaded on the surface of dendritic cell which activates cytotoxic T cells responsible for specific anti-tumor response. After immunogenically-killed cells DC stimulation the emission of DEX could be observed. So, the DEX-based immunization implies collecting this ICD-mediated dexosomes and using them as an anti-tumor vaccine.

ICD proprieties suggest that immunogenically killed cells could be applied in the production of DC-derived exosomes, as this strategy could potentially enhance the therapeutic efficacy of extracellular vesicles (**Figure 4**). The content of DEXs is well characterized and was discussed earlier in this review. However, the impact of immunogenically killed cells on the resulting ICD-DEX remains largely unexplored, highlighting the need for further studies.

### 3.7 Challenges and strategies to enhance the anti-tumor efficacy of dendritic cell-derived exosomes in cancer immunotherapy

Dexosomes promote a strong T cell response in both *in vitro* and *in vivo* models. DEXs are often described as vesicles with strong antigen-presenting properties, yet DEX immunotherapy faces challenges in clinical trials and treatment integration (92, 120). Most clinical trials listed in databases do not progress to the next phases or are even terminated without providing results (88), and clinical applications seem to have stuck in a holding pattern. On the other hand, fundamental research has explored DEX properties, content, and their so-called phenotype, or surface molecule profile, in depth. Therefore, this matter calls for explanation.

First, DEXs are not living cells, so they cannot actively migrate to the tumor or lymph nodes like DCs. Instead, exosomes are transported from peripheral tissues through lymphatic vessels and nodes in complex with lymphatic endothelial cells (149). Second, the complexity of DEX composition can vary significantly, making it difficult to standardize the treatment and manufacturing process (150). Interestingly, artificially generated DC subtypes, e.g. cDC obtained after PIB-induced transformation, might become a prospective source of DEX in context of both routine and novel manufacturing designs. Furthermore, dendritic cells subtypes obtained with INF-γ simulation show solid efficacy and immunogenicity. Exosomes derived from DC stimulated with IFN-γ show imposing therapeutic effect in combination with chemotherapeutic treatment of non-small cell lung cancer. Controlled stimulation of dendritic cells phenotypes might become one of the solutions on the way to standardization of DEX manufacturing and immunogenicity enhancement of therapy (151–153). Still discussing the possibility of using PIB-induced or other derived DC is important in context of the ability to use them as full-fledged alternative of DEX source as this transformation strategy might face the problems of incomplete functional maturation, variable reprogramming efficiency, reduced proliferative activity, and genetic and epigenetic barriers (154). DEX are easy to store and transport, but they still originate from DCs derived from live donors or cancer patients, which requires complex invasive procedures. The ‘quality’ of the original biospecimens and the source and subtypes of DCs also play an important role in the treatment success (155). The last limitation we would like to highlight is the lack of pre-selection criteria. There is currently no standardized set of criteria for selecting patients who would benefit most from DEX therapy, which complicates the identification of optimal candidates (156). This is particularly evident in clinical trials when the immune backgrounds of patients and treatment strategies differ a lot in each case.

All these limitations of DEXs necessitate modifications to enhance their anti-tumor properties. Therefore, researchers propose strategies to address these challenges and improve existing methods. DEXs are more compatible with different modifications like genetic engineering, surface treatment, and exogenous cargo loading. In particular, genetic engineering enables target ligand expression and can be employed as a therapeutic strategy. For instance, overexpression of several proteins in precursor DCs results in CD8+ reprogramming and an enhanced CTL response following DEX treatment. Another approach is to inhibit the PD-1/PD-L1 pathway, which tumors exploit to evade immune detection. PD-1 is a T-cell checkpoint protein engaged by its ligand PD-L1 that inhibits T-cell activation. Parental DCs transfected with a plasmid can successfully express anti-PD-1 antibodies. Alternatively, DEXs can be loaded with anti-PD-1 antibodies. Thus, anti-PD-1 antibodies in combination with DEXs can significantly enhance T cell activity against tumors (92, 157). Additionally, antigens or adjuvant molecules can be directly conjugated to the cell surface. As demonstrated in macrophages, such a modification could be utilized to modify DEX in future research (158). Preconditioning DCs with cytokines, such as IFN-γ, can enhance the immunogenicity of the resulting exosomes. IFN-γ preconditioning promotes DC maturation and increases the pro-inflammatory cytokine production, which can improve the efficacy of DEXs against tumors (150). DEXs can be loaded with specific molecules endogenously by using viral vectors or plasmids to modify parental cells (159).

## 4 Conclusion

Over 25 years, since the first description of DEX functions and pilot *in vivo* experiments, DC-derived exosomes are still under active research focus. DEXs have the potential to control, suppress, and target tumors *in vitro* and *in vivo*. Due to these properties, DEX are therapeutically promising cell-derived elements that can efficiently present antigens, overcome immunosuppression in the tumor microenvironment, and enhance immunogenicity (85, 89, 98). In addition, although DEX-based drugs have reached the stage of clinical trials, they were not successful for a number of reasons. DEXs are cell-derived products rather than viable cells. The main benefit of DEX-based therapy is that the exosomes cannot be recruited by immune cells in the tumor microenvironment. Furthermore, some studies show that they can reprogram host’s immune cells previously recruited by tumors. However, despite all DEX benefits, there is great room for further research and improvements. For example, it has never been described how the cells that died via the ICD pathway impact the efficacy of the resulting exosomes. We propose that DEX obtained following an ICD stimulus can promote a significantly more effective response against a tumor, as demonstrated by DC vaccines in a variety of murine models (57, 160–162). The ICD-DEX approach requires further study and validation, both *in vitro* and *in vivo*. If successful, it could become an appealing option for clinical practice, not only as a monotherapy or a part of multicomponent treatment but also as an agent that enhances the immune response through its impact on the tumor microenvironment.
